# Evaluation of von Willebrand factor in COPD patients*

**DOI:** 10.1590/S1806-37132014000400004

**Published:** 2014

**Authors:** Thiago Prudente Bártholo, Cláudia Henrique da Costa, Rogério Rufino

**Affiliations:** Rio de Janeiro State University, Rio de Janeiro, Brazil; Department of Pulmonology, Rio de Janeiro State University, Rio de Janeiro, Brazil; Department of Pulmonology, Rio de Janeiro State University, Rio de Janeiro, Brazil

**Keywords:** von Willebrand factor, Pulmonary disease, chronic obstructive, Endothelial cells

## Abstract

**OBJECTIVE:**

To compare the absolute serum von Willebrand factor (vWF) levels and relative serum vWF activity in patients with clinically stable COPD, smokers without airway obstruction, and healthy never-smokers.

**METHODS:**

The study included 57 subjects, in three groups: COPD (n = 36); smoker (n = 12); and control (n = 9). During the selection phase, all participants underwent chest X-rays, spirometry, and blood testing. Absolute serum vWF levels and relative serum vWF activity were obtained by turbidimetry and ELISA, respectively. The modified Medical Research Council scale (cut-off score = 2) was used in order to classify COPD patients as symptomatic or mildly symptomatic/asymptomatic.

**RESULTS:**

Absolute vWF levels were significantly lower in the control group than in the smoker and COPD groups: 989 ± 436 pg/mL vs. 2,220 ± 746 pg/mL (p < 0.001) and 1,865 ± 592 pg/mL (p < 0.01). Relative serum vWF activity was significantly higher in the COPD group than in the smoker group (136.7 ± 46.0% vs. 92.8 ± 34.0%; p < 0.05), as well as being significantly higher in the symptomatic COPD subgroup than in the mildly symptomatic/asymptomatic COPD subgroup (154 ± 48% vs. 119 ± 8%; p < 0.05). In all three groups, there was a negative correlation between FEV_1_ (% of predicted) and relative serum vWF activity (r^2^ = −0.13; p = 0.009).

**CONCLUSIONS:**

Our results suggest that increases in vWF levels and activity contribute to the persistence of systemic inflammation, as well as increasing cardiovascular risk, in COPD patients.

## Introduction

Worldwide, COPD is a public health problem, affecting more than 10% of the population over the age of 50 years.^(^ 1 ^,^ 2 ^)^ The prevalence of this disease has increased particularly in developing countries.^(^ 3 ^)^ It is estimated that, in 2020, COPD will be the third leading cause of death worldwide. This obstructive disease is usually associated with smoking,^(^ 3 ^)^ and COPD patients are at a higher risk of cardiovascular changes than is the general population.^(^ 4 ^,^ 5 ^)^

Recently, the presence of a systemic inflammation process has been found to be associated with some complications in COPD patients, chief among which are cachexia, anorexia, osteoporosis, and atherosclerosis.^(^ 2 ^,^ 6 ^)^ However, it has yet to be clearly established whether comorbidities are a consequence of lung disease or whether COPD can be considered a systemic disease. Inflammation is believed to also occur at the endothelial level, contributing to the formation of atherosclerotic plaques.^(^ 7 ^)^ This vascular event could partially explain the higher prevalence of cardiovascular diseases in smokers who develop airway obstruction.^(^ 7 ^)^ Some inflammatory and endothelial markers, such as C-reactive protein and fibrinogen, are increased in COPD patients.^(^ 7 ^,^ 8 ^)^ Von Willebrand factor (vWF) is a marker of endothelial damage and participates in the process of atherosclerosis.^(^ 9 ^)^ Increased serum vWF levels have been reported in COPD patients during exacerbations.^(^ 10 ^)^ The objective of the present study was to assess the behavior of vWF levels in stable COPD patients who had not experienced a recent exacerbation, as well as attempting to correlate this endothelial marker with respiratory disease severity.

## Methods

The present study was approved by the local research ethics committee, and all participants gave written informed consent before undergoing any study procedures. In addition, this project was in compliance with current ethics regulations in Brazil.

Patients were selected from among those under follow-up at the outpatient clinic of the Department of Pulmonology and Tuberculosis of the Rio de Janeiro State University, located in the city of Rio de Janeiro, Brazil, and professionals working at that clinic were invited to participate as volunteers. Between February of 2011 and July of 2012, a total of 57 subjects were recruited in three groups: COPD; smoker; and control. The inclusion criteria for the group of COPD patients were having a smoking history of at least 20 pack-years and having a post-bronchodilator FEV_1_/FVC ratio < 0.7. Smokers should also have a long smoking history (at least 20 pack-years), but they should have normal spirometry results at selection. Healthy volunteers should have no history of lung disease, should be never-smokers, and should have normal spirometry results. The exclusion criteria for the three groups were as follows: having a history of asthma, atopy, or atherosclerotic cardiovascular disease; having had respiratory infection in the last three weeks; having recently been diagnosed with or being under treatment for tuberculosis; having congestive heart failure, HIV infection, diseases that are systemic and inflammatory in origin, severe dyslipidemia (serum triglyceride levels > 300 mg/dL or total cholesterol levels > 280 mg/dL), and diabetes mellitus (diagnosed in accordance with the American Diabetes Association criteria)^(^ 11 ^)^; having used systemic anti-inflammatory agents or antiplatelet drugs regularly in the last year; and having abnormal laboratory test results at selection. Patients with COPD should be using their usual medications and should not have experienced exacerbations of their disease for at least three months. During the selection phase, ancillary tests included spirometry, chest X-rays, and blood testing. Spirometry was performed with a Vitatrace spirometer (Pró Médico Ltda., Rio de Janeiro, Brazil), in accordance with the American Thoracic Society standards,^(^ 12 ^)^ and all subjects underwent bronchodilator testing with albuterol (400 µg). The reference equations of Pereira et al. were used.^(^ 13 ^)^ Blood testing included blood workup, coagulation profile, and determination of serum glucose, urea, creatinine, uric acid, triglyceride, total cholesterol, and HDL/LDL cholesterol levels. For the selected subjects only, a blood sample was stored at −80°C and sent for analysis of absolute vWF levels (turbidimetry) and relative serum vWF activity (ELISA). Chest X-rays were performed on the same day as spirometry and blood sample collection. The X-rays were examined by a radiologist and were used in patient selection, because healthy volunteers and smokers should not have radiographic changes. Patients with COPD often had small scarring suggestive of a history of tuberculosis or signs of hyperinflation. Patients with other X-ray findings, especially when associated with clinical changes suggesting active disease, were excluded from the study.

All 57 recruited subjects met the inclusion criteria and met none of the exclusion criteria. Of those, 36 had a diagnosis of COPD, 12 were smokers without airflow obstruction, and 9 were healthy volunteers.

Classification of COPD was based on the Global Initiative for Chronic Obstructive Lung Disease (GOLD) strategy document.^(^ 14 ^)^ Therefore, symptoms and number of exacerbations of the disease in the previous year were identified and, together with post-bronchodilator measurement of FEV_1_ (% of predicted), were used to assign patients to categories A, B, C, or D. Symptoms were quantified with the modified Medical Research Council (mMRC) scale, whose scores are used to determine the presence or absence of symptoms (mMRC score ≥ 2 and mMRC score < 2, respectively).^(^ 14 ^)^ On this basis, 13, 5, 7, and 11 of the 36 COPD patients were classified as belonging to subgroups A, B, C, and D, respectively. According to the spirometric classification, without considering symptoms or the presence of exacerbations, 11 patients had mild COPD, 13 had moderate COPD, and 12 had severe COPD.

Statistical analysis was performed with the GraphPad Prism software, version 6 (GraphPad Software Inc., San Diego, CA, USA). ANOVA and Dunn's post hoc test were used to compare groups, and the Mann-Whitney test was used to compare independent groups. Nonparametric Spearman's test was used to compare two variables. The level of significance was set at p < 0.05.

## Results

Of the 57 subjects recruited, 31 were male. Age was significantly higher in the COPD group than in the other two groups, whereas it was similar in the control and smoker groups. Spirometric data for the groups are shown in Table 1. Comorbidities were found in all three groups; however, they were more common in the COPD group (Table 1). 

**Table 1:** Demographic and spirometric data of the study participants.a

Variables	Groups		
	Control	Smoker	COPD
	(n = 9)	(n = 12)	(n = 36)
Age, years	47.22 ± 1.41	50.30 ± 4.94	62.75 ± 9.98
Male/Female, n/n	4/5	3/9	24/12
FVC, L	3.37 ± 1.20	3.38 ± 0.61	2.90 ± 0.95
FVC, % of predicted	100.88 ± 12.17	103.30 ± 12.10	86.08 ± 20.23
FEV_1_, L	2.98 ± 0.72	2.78 ± 0.54	1.59 ± 0.69
FEV_1_,% of predicted	99.31 ± 11.02	104 ± 9.87	59.84 ± 21.30
FEV_1_/FVC, %	79.67 ± 5.19	83.90 ± 9.68	53.07 ± 10.54
Comorbidities^b^			
SAH	1	3	9
Hypothyroidism		2	1
Dyslipidemia		1	
Glaucoma			1
Bipolar disorder			1
Calcinosis			1

SAH : systemic arterial hypertension

a Values expressed as mean ± SD, except where otherwise indicated

b Values expressed as n of patients.

**Figure 1:**
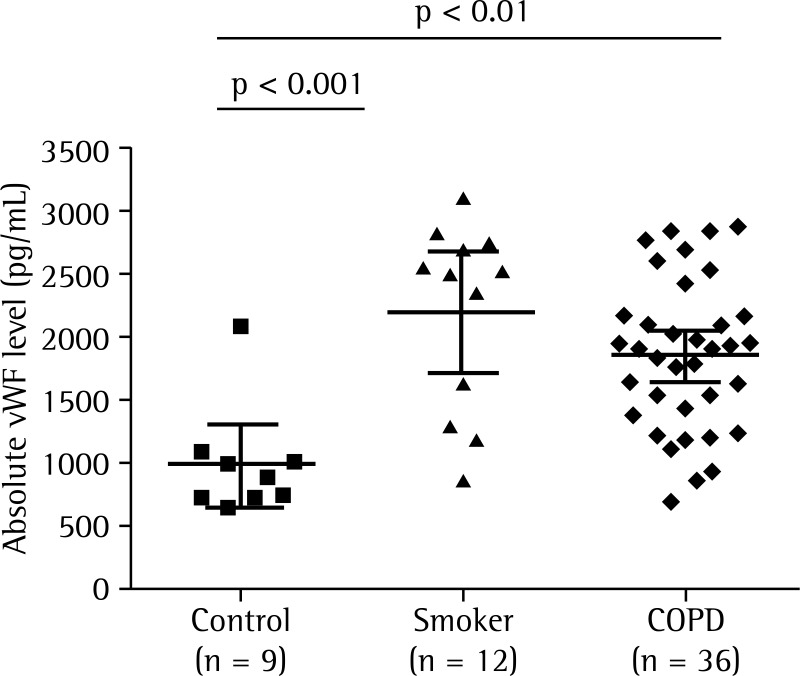
Absolute serum von Willebrand factor (vWF) levels in the groups studied.

**Figure 2:**
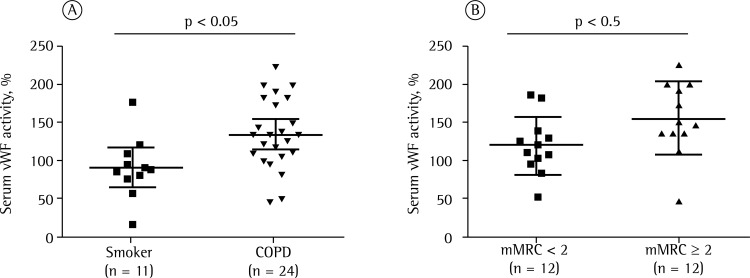
Relative serum von Willebrand factor (vWF) activity. In A, comparison between the smoker and COPD groups. In B, comparison between the symptomatic COPD and mildly symptomatic/asymptomatic COPD subgroups as defined by the modified Medical Research Council (mMRC) scale scores.

Serum vWF levels were measured by two different methods. The first determined absolute serum vWF levels. The control group had significantly lower absolute vWF levels than did the smoker and COPD groups: 989 ± 436 pg/mL vs. 2,220 ± 746 pg/mL (p < 0.001) and 1,865 ± 592 pg/mL (p < 0.01), respectively (Figure 1). The second method used determined relative serum vWF activity. The COPD group had significantly higher values than did the smoker group (136.7 ± 46.0% vs. 92.8 ± 34.0%; p < 0.05; Figure 2A).

In order to assess the relationship between serum vWF levels and COPD severity, we subdivided the COPD group into four categories, i.e., GOLD groups A, B, C, and D.^(^ 14 ^)^ However, neither absolute serum levels nor relative serum activity showed correlations with this classification. Likewise, we found no correlation of absolute serum vWF levels or relative serum vWF activity with the spirometric classification of COPD. The ANOVA did not allow us to distinguish among the four subgroups of patients on the basis of absolute vWF levels or relative serum vWF activity (p > 0.05). The 18 patients classified as GOLD group C or D were using inhaled corticosteroids, because this is the treatment approach used at out facility. No correlation was found between inhaled corticosteroid use and absolute serum vWF levels or relative serum vWF activity.

In a second analysis, COPD patients were subdivided into two groups on the basis of their level of dyspnea as measured by the mMRC scale. Patients with an mMRC score ≥ 2 were considered symptomatic. In this analysis, there was no significant difference in absolute vWF levels between the symptomatic and mildly symptomatic/asymptomatic groups. However, relative serum vWF activity was significantly higher in the symptomatic group than in the mildly symptomatic/asymptomatic group (154.0 ± 48.0% vs. 118.9 ± 38.0%; p < 0.05; Figure 2B)

Subsequently, COPD patients were further subdivided into two groups on the basis of the presence or absence of exacerbations (presence being defined as ≥ 2 exacerbations in the last year and absence being defined as < 2 exacerbation in the last year). There were no significant differences in absolute serum vWF levels or relative serum vWF activity between the two subgroups.

**Figure 3:**
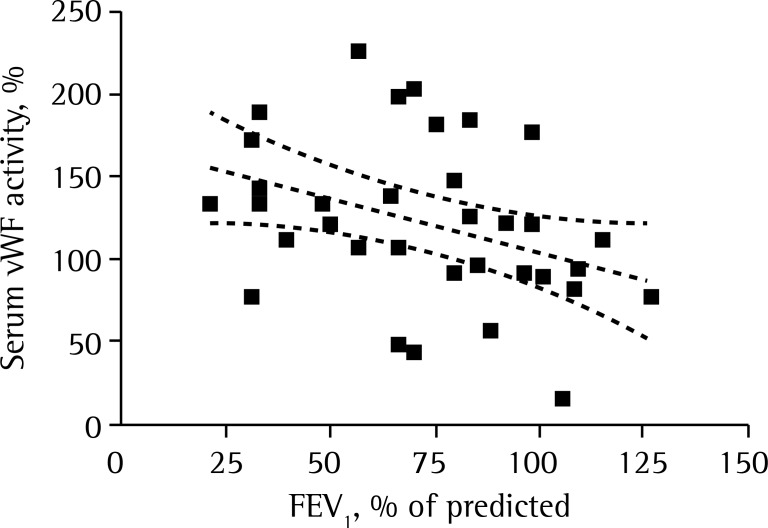
Relationship between relative serum von Willebrand factor (vWF) activity and FEV1 (% of predicted; r2 = -0.13; p = 0.0099).

In the control, smoker, and COPD groups, there was a significant negative correlation between FEV_1_ (% of predicted) and relative serum vWF activity (r^2^ = −0.13; p = 0.009; Figure 3), whereas there was no correlation between FEV_1_ (% of predicted) and absolute vWF levels (p = 0.077).

## Discussion

The fourth leading cause of death worldwide, COPD affects approximately 16% of the population in the city of São Paulo, Brazil.^(^ 15 ^)^ One study demonstrated that COPD is underdiagnosed in this city, because 83% of the subjects with airway obstruction did not have a clinical diagnosis of COPD.^(^ 16 ^)^ This scenario remains virtually unchanged, as shown in a 9-year follow-up study, which found that 70% of the respondents had obstruction as diagnosed by spirometry.^(^ 16 ^)^ In addition to destroying the alveolar septa, COPD seems to have a systemic inflammatory effect.^(^ 17 ^)^ It is possible that this inflammation also affects the endothelial system,^(^ 7 ^)^ the impairment of which could partially explain the high prevalence of vascular disease in COPD patients. Some studies have attempted to relate increased levels of some endothelial markers, such C-reactive protein and fibrinogen, to COPD.^(^ 7 ^,^ 8 ^)^ One study reported increased vWF levels in COPD patients during exacerbations.^(^ 10 ^)^ However, the role of this marker in COPD during the stable phase of the disease has yet to be established. The vWF can be evaluated in two different ways: by measurement of its absolute serum levels and by measurement of its relative serum activity. The first is a quantitative evaluation, whereas the second leads us to a qualitative analysis.

In the present study, the authors found that absolute serum vWF levels were higher in smokers (with and without airflow obstruction) than in controls (p < 0.01). The relationship between smoking and increased vWF levels has been demonstrated in recent years, there seeming to be a significant increase of up to 76% in vWF levels after 120 minutes of tobacco use, as well as an average decrease from 144% to 123% in vWF levels in patients who quit smoking.^(^ 8 ^)^ One study demonstrated that vWF activity is increased in smokers.^(^ 18 ^)^ One group of authors reported that vWF levels are higher in COPD patients than in healthy subjects; however, smokers without obstruction were not included in that analysis.^(^ 19 ^)^ Another study demonstrated that serum vWF levels increase in COPD patients during exacerbations.^(^ 20 ^)^ In the present study, the presence of an exacerbation was considered an exclusion criterion, because our objective was to analyze vWF levels during the stable phase of COPD. Therefore, it was impossible to determine any association with that variable. The increase in relative vWF activity in COPD patients, when compared with the smoker group, suggests that vWF may play a role in the inflammatory pathophysiology of COPD and could be related to atherosclerosis and cardiovascular disease.^(^ 7 ^)^

To our knowledge, the present study is the first to attempt to correlate vWF levels with COPD severity as defined by the GOLD classification.^(^ 14 ^)^ However, no statistically significant difference was found in serum vWF levels among the four COPD severity groups, nor were there any differences among the groups when the spirometric classification of COPD was considered. This suggests that, although vWF levels are high in stable COPD patients, they do not correlate with disease severity. This finding is consistent with literature reports that relate vWF levels to other inflammatory diseases, such as diabetes mellitus and rheumatoid arthritis.^(^ 10 ^,^ 21 ^)^ It seems that vWF is a nonspecific marker of inflammation, and therefore it is not useful to grade the severity of chronic inflammatory diseases.

When we used the mMRC scale to determine the presence or absence of symptoms, we found that relative serum vWF activity was significantly higher in symptomatic patients, i.e., those with an mMRC score ≥ 2 (p < 0.05). This possibly indicates that the degree of inflammation is higher in symptomatic patients than in mildly symptomatic or asymptomatic patients. Following this line of reasoning, it was expected that patients with frequent exacerbations would have higher vWF levels, which was not observed in the present sample. Thus, further studies are needed to elucidate this issue.

Although there was a significant negative correlation between FEV_1_ (% of predicted) and relative serum vWF activity in all three groups (control, smoker, and COPD), the correlation was not very robust (Figure 3). In addition, one study found no correlations between vWF levels and decline in FEV_1_.^(^ 22 ^)^ Therefore, studies involving a larger number of patients are needed to clarify this issue.

The present study has some limitations, chief among which is the fact that the control and smoker groups were not matched for age with the COPD group, which is something very difficult to achieve in studies that compare patients with and without bronchial obstruction. However, healthy volunteers (controls) and smokers were similar in age. Nevertheless, vWF levels were significantly higher in the smoker group. Another important fact is that participants were not screened for blood group (ABO blood typing system), and blood group has a small influence on vWF levels. A third limitation was the lack of evaluation of other inflammatory parameters, such as C-reactive protein and fibrinogen. This evaluation would allow us to analyze them in comparison with related data in the literature and with serum vWF levels. In contrast, an attempt was made to exclude a large number of factors that could be related to systemic inflammation and endothelial injury. Thus, as reported in Methods, patients or volunteers with a history of cardiovascular disease or other chronic or infectious diseases, as well as those who were using medications, were excluded from the study, and this considerably limited the recruitment of participants.

Patients with COPD are at a higher risk of endothelial injury and consequent cardiovascular disease. In our study, absolute serum vWF levels were higher in smokers with and without bronchial obstruction than in controls, and relative serum vWF activity was higher in COPD patients than in smokers. It is possible that vWF participates in the systemic inflammatory process in COPD patients and thereby contributes to increasing cardiovascular risk.
