# Supplementary material for: Evaluation of von Willebrand factor in COPD patients
Source: J Bras Pneumol. 2014 Jul-Aug;40(4):373–9. doi: 10.1590/S1806-37132014000400004 (PMC4201167; doi:10.1590/S1806-37132014000400004)
Supplement: Translation

# Translation: Avaliação do fator de von Willebrand em pacientes com DPOC*

**Authors:** Thiago Prudente Bártholo, Cláudia Henrique da Costa, Rogério Rufino

**Affiliations:** Universidade do Estado do Rio de Janeiro, Rio de Janeiro (RJ) Brasil; Disciplina de Pneumologia, Universidade do Estado do Rio de Janeiro, Rio de Janeiro (RJ) Brasil; Disciplina de Pneumologia, Universidade do Estado do Rio de Janeiro, Rio de Janeiro (RJ) Brasil

**Keywords:** Fator de von Willebrand, Doença pulmonar obstrutiva crônica, Células endoteliais

## Abstract

**OBJETIVO:**

Comparar os níveis séricos absolutos e a atividade sérica em percentual do fator de von Willebrand (FvW) em pacientes com DPOC clinicamente estáveis, tabagistas sem obstrução das vias aéreas e em indivíduos saudáveis que nunca fumaram.

**MÉTODOS:**

Foram incluídos no estudo 57 indivíduos, em três grupos: DPOC (n = 36), tabagista (n = 12) e controle (n = 9). Todos os participantes realizaram radiografia do tórax, espirometria e exame de sangue durante a fase de seleção. Os níveis séricos absolutos e a atividade sérica em percentual do FvW foram obtidos por turbidimetria e ELISA, respectivamente. A escala *Medical Research Council* modificada foi utilizada para classificar pacientes como sintomáticos ou assintomáticos/pouco sintomáticos no grupo DPOC (ponto de corte = 2).

**RESULTADOS:**

Os níveis absolutos do FvW no grupo controle foram significativamente menores que os nos grupos tabagista e DPOC: 989 ± 436 pg/mL vs. 2.220 ± 746 pg/mL (p < 0,001) e 1.865 ± 592 pg/mL (p < 0,01). Os valores em percentual de atividade do FvW no grupo DPOC foram significativamente maiores que no grupo tabagista (136,7 ± 46,0% vs. 92,8 ± 34,0%; p < 0,05), assim como foram significativamente maiores no subgrupo DPOC sintomático que no subgrupo DPOC assintomático/pouco sintomático (154 ± 48% vs. 119 ± 8%; p < 0,05). Houve uma correlação negativa entre o VEF_1_ (% do previsto) e os níveis em percentual de atividade do FvW nos três grupos (r^2^ = −0,13; p = 0,009).

**CONCLUSÕES:**

Nossos resultados sugerem que aumentos nos níveis de FvW e de sua atividade contribuem para a manutenção da inflamação sistêmica e o aumento do risco cardiovascular em pacientes com DPOC.

## Introdução

A DPOC é um problema de saúde pública em todo o mundo acometendo mais de 10% da população acima de 50 anos.^(^ 1 ^,^ 2 ^)^ A prevalência dessa doença tem aumentado particularmente nos países em desenvolvimento.^(^ 3 ^)^ Estima-se que em 2020 a DPOC será a terceira principal causa de mortalidade em todo o mundo. Essa doença obstrutiva costuma estar relacionada ao hábito de fumar,^(^ 3 ^)^ e os pacientes apresentam um risco maior de alterações cardiovasculares quando comparados à população em geral.^(^ 4 ^,^ 5 ^)^

Recentemente, verificou-se que a presença de um processo de inflamação sistêmico tem sido associada a algumas complicações dos pacientes com DPOC, destacando-se a caquexia, a anorexia, a osteoporose e a aterosclerose.^(^ 2 ^,^ 6 ^)^ Entretanto, ainda não está bem estabelecido se as comorbidades são consequências da doença pulmonar ou se a DPOC pode ser considerada uma doença sistêmica. Acredita-se que a inflamação ocorra também ao nível endotelial, concorrendo para a formação de placas ateroscleróticas.^(^ 7 ^)^ Esse evento vascular poderia, em parte, explicar a maior prevalência de doenças cardiovasculares em pacientes tabagistas que evoluem com obstrução das vias aéreas.^(^ 7 ^)^ Alguns marcadores inflamatórios e endoteliais, como a proteína C reativa e o fibrinogênio, estão aumentados nos pacientes com DPOC.^(^ 7 ^,^ 8 ^)^ O fator de von Willebrand (FvW) é um marcador de dano endotelial e participa do processo de aterosclerose.^(^ 9 ^)^ Já foi relatado o aumento sérico desse fator em pacientes com DPOC durante a fase de exacerbação.^(^ 10 ^)^ O objetivo do presente estudo foi avaliar o comportamento dos níveis de FvW em pacientes com DPOC, estáveis e sem exacerbação recente e, ainda, tentar correlacionar esse marcador endotelial com a gravidade da doença respiratória.

## Métodos

O presente estudo foi aprovado pelo comitê de ética em pesquisa da instituição, e todos os participantes assinaram o termo de consentimento livre e esclarecido antes de realizar qualquer procedimento para o estudo, estando o presente projeto de acordo com a regulação ética vigente no país.

Foram selecionados pacientes em acompanhamento no ambulatório da Disciplina de Pneumologia e Tisiologia da Universidade do Estado do Rio de Janeiro, localizado na cidade do Rio de Janeiro (RJ), e profissionais que trabalham no ambulatório foram convidados a participar como voluntários. No total, foram recrutados 57 indivíduos entre fevereiro de 2011 e julho de 2012, em três grupos: DPOC, tabagista e controle. Os critérios de inclusão para o grupo de pacientes com DPOC foram ter carga tabágica de pelo menos 20 maços-ano e apresentar uma relação VEF_1_/CVF após o uso de broncodilatador < 0,7. Os indivíduos tabagistas também deveriam apresentar carga tabágica alta, de pelo menos 20 maços-ano, mas o resultado da espirometria realizada na seleção dos indivíduos deveria ser normal. Os voluntários sadios não poderiam ter nenhum histórico de doença pulmonar, nunca ter fumado e apresentar espirometria normal. Os critérios de exclusão para os três grupos foram apresentar história de asma, atopia ou doença cardiovascular aterosclerótica; ter tido infecção respiratória nas últimas três semanas; apresentar tuberculose recém diagnosticada ou em tratamento; apresentar insuficiência cardíaca congestiva, infecção por HIV, patologias com origem inflamatória sistêmica, dislipidemia grave (níveis séricos de triglicerídeos > 300 mg/dL ou colesterol total > 280 mg/dL), diabetes mellitus (diagnosticada de acordo com os critérios da *American Diabetes Association*)^(^ 11 ^)^; fazer uso regular de anti-inflamatórios sistêmicos ou de drogas antiplaquetárias no último ano; e apresentar alterações nos exames laboratoriais na fase de seleção. Os pacientes com DPOC deveriam estar em uso de seus medicamentos usuais, sem haver apresentado exacerbações da doença por pelo menos três meses. Os exames complementares realizados durante a seleção foram espirometria, radiografia de tórax e exame de sangue. A espirometria foi realizada com um espirômetro Vitatrace (Pró Médico Ltda., Rio de Janeiro, Brasil) seguindo a padronização da American *Thoracic Society*,^(^ 12 ^)^ e todos os indivíduos realizaram prova broncodilatadora com salbutamol (400 µg). As equações de referência utilizadas foram as de Pereira et al.^(^ 13 ^)^ O exame de sangue incluiu hemograma completo, coagulograma e dosagens séricas de glicose, ureia, creatinina, ácido úrico, triglicerídeos e colesterol total e frações. Apenas para os indivíduos selecionados, uma amostra de sangue foi estocada a −80°C e enviada para a análise dos níveis absolutos de FvW por turbidimetria e da proporção de atividade sérica do FvW por ELISA. As radiografias de tórax foram obtidas no mesmo dia da realização da espirometria e da coleta de amostra de sangue. As radiografias foram examinadas por um radiologista e foram utilizadas na seleção de pacientes, pois os voluntários saudáveis e tabagistas não podiam apresentar alterações. Os pacientes com DPOC frequentemente apresentavam pequenas lesões cicatriciais sugestivas de história de tuberculose ou sinais de hiperinsuflação. Os pacientes que apresentavam outros achados radiográficos, especialmente quando associados a alterações clínicas sugerindo doenças ativas, foram excluídos do estudo.

Todos os 57 indivíduos recrutados cumpriram os critérios de inclusão e nenhum critério de exclusão. Desses, 36 apresentavam diagnóstico de DPOC, 12 eram fumantes sem obstrução ao fluxo aéreo, e 9 eram voluntários sadios.

A classificação da DPOC foi realizada de acordo com as recomendações do documento da *Global Initiative for Chronic Obstructive Lung Disease* (GOLD).^(^ 14 ^)^ Assim, os sintomas e o número de exacerbações da doença no ano anterior foram verificados e, junto com a mensuração do VEF_1_ após o uso de broncodilatador em percentual do previsto, foram utilizados para alocar os pacientes nas categorias A, B, C ou D. Os sintomas foram quantificados de acordo com a escala do *Medical Research Council* modificada (MRCm), pela qual se define a presença ou a ausência de sintomas (MRCm ≥ 2 e MRCm < 2, respectivamente).^(^ 14 ^)^ A partir dessa classificação, dos 36 pacientes com DPOC, 13, 5, 7 e 11 pacientes foram classificados, respectivamente, nos subgrupos A, B, C e D. Ao analisarmos a classificação espirométrica sem considerar os sintomas ou a presença de exacerbações, 11 pacientes apresentavam DPOC leve, 13 apresentavam DPOC moderada, e 12 pacientes apresentavam DPOC grave.

A avaliação estatística foi realizada utilizando o programa GraphPad Prism, versão 6 (GraphPad Software Inc., San Diego, CA, EUA). ANOVA e teste *post hoc* de Dunn foram utilizados para a comparação de grupos. O teste de Mann-Whitney foi utilizado para a comparação de grupos independentes. A análise comparativa de duas variáveis foi realizada através do teste não paramétrico de Spearman. O nível de significância estabelecido foi de p < 0,05.

## Resultados

Dos 57 indivíduos recrutados, 31 eram do sexo masculino. Os pacientes do grupo DPOC significativamente apresentavam idade mais avançada do que os participantes dos outros dois grupos, enquanto controles e tabagistas apresentavam idades similares. Os dados espirométricos nos grupos são apresentados na Tabela 1. A presença de comorbidades foi encontrada em todos os grupos; entretanto, no grupo DPOC, essas foram mais frequentes (Tabela 1).

**Tabela 1:** Dados demográficos e espirométricos dos participantes do estudo.a

Variáveis	Grupos		
	Controle	Tabagista	DPOC
	(n = 9)	(n = 12)	(n = 36)
Idade, anos	47,22 ± 1,41	50,30 ± 4,94	62,75 ± 9,98
M/F, n/n	4/5	3/9	24/12
CVF, L	3,37 ± 1,20	3,38 ± 0,61	2,90 ± 0,95
CVF, % previsto	100,88 ± 12,17	103,30 ± 12,10	86,08 ± 20,23
VEF_1_, L	2,98 ± 0,72	2,78 ± 0,54	1,59 ± 0,69
VEF_1_,% previsto	99,31 ± 11,02	104 ± 9,87	59,84 ± 21,30
VEF_1_/CVF, %	79,67 ± 5,19	83,90 ± 9,68	53,07 ± 10,54
Comorbidades^b^			
HAS	1	3	9
Hipotireoidismo		2	1
Dislipidemia		1	
Glaucoma			1
Distúrbio bipolar			1
Calcinose			1

M/F : masculino/feminino

HAS : hipertensão arterial sistêmica

a Valores expressos em média ± dp, exceto onde indicado

b Valores expressos em n de pacientes.

Os níveis séricos do FvW foram obtidos através de duas metodologias distintas. A primeira avaliou o nível sérico absoluto desse fator. O grupo controle apresentou níveis absolutos de FvW significativamente menores que os dos grupos tabagista e DPOC: 989 ± 436 pg/mL vs. 2.220 ± 746 pg/mL (p < 0,001) e 1.865 ± 592 pg/mL (p < 0,01), respectivamente (Figura 1). A segunda metodologia utilizada verificou a proporção de atividade sérica do FvW. Os pacientes do grupo DPOC apresentaram valores significativamente maiores do que os do grupo tabagista (136,7 ± 46,0% vs. 92,8 ± 34,0%; p < 0,05; Figura 2A).

**Figura 1:** Níveis séricos absolutos do fator de Von Willebrand (FvW) nos grupos estudados.

**Figura 2:** Níveis séricos do percentual de atividade do fator de Von Willebrand (FvW). Em A, comparação entre os grupos tabagista e DPOC. Em B, comparação entre os subgrupos DPOC sintomáticos e DPOC assintomáticos/pouco sintomáticos de acordo com os escores da escala Medical Research Council modificada (MRCm).

Com o objetivo de avaliar a relação entre o nível sérico do FvW e a gravidade da DPOC, o grupo DPOC foi subdividido em quatro diferentes categorias, de acordo com os níveis de classificação GOLD A, B, C e D.^(^ 14 ^)^ Entretanto, nem o nível sérico absoluto e nem o percentual de atividade sérica demonstraram relações com essa classificação da doença. Da mesma forma, também não verificamos correlações dos níveis séricos absolutos e dos percentuais de atividade sérica do FvW com a classificação espirométrica da DPOC. Utilizando ANOVA, não foi possível diferenciar esses quatro subgrupos de pacientes pelos níveis absolutos ou percentuais de atividade do FvW (p > 0,05). Os 18 pacientes classificados como GOLD C ou D estavam em uso de corticoide inalatório, pois esta é a conduta em nosso serviço. Também não foi verificada uma relação dos níveis séricos absolutos ou percentuais do FvW com o uso de corticoide inalatório.

Em uma segunda análise, os pacientes com DPOC foram subdivididos em dois diferentes grupos, de acordo com o grau de dispneia com base na escala MRCm. Consideramos os pacientes com pontuação na escala de MRCm ≥ 2 como sintomáticos. Nessa análise, não houve uma diferença significativa do valor absoluto de FvW entre os grupos sintomático e assintomático/pouco sintomático. Entretanto, o percentual de atividade sérica do FvW no grupo sintomático foi significativamente maior do que aquele no grupo assintomático/pouco sintomático (154,0 ± 48,0% vs. 118,9 ± 38,0%; p < 0,05; Figura 2B).

Posteriormente, os pacientes com DPOC foram novamente subdivididos em dois grupos distintos de acordo com a presença (≥ 2 exacerbações no último ano) ou ausência de exacerbações (< 2 de exacerbações no último ano). Não houve diferenças significativas dos valores absolutos ou percentuais do FvW entre os dois subgrupos.

Foi encontrada uma correlação negativa significativa dos valores de VEF_1_ em porcentagem do previsto com os níveis percentuais de atividade sérica do FvW entre os grupos controle, tabagista e DPOC (r^2^ = −0,13; p = 0,009; Figura 3), enquanto não houve uma correlação entre os valores de VEF_1_ em porcentagem do previsto com os níveis absolutos do FvW entre os grupos (p = 0,077).

**Figura 3:** Relação entre os valores do percentual de atividade do fator de Von Willebrand (FvW) e VEF1 em porcentagem do previsto (r2 = -0,13; p = 0,0099).

## Discussão

A DPOC é a quarta principal causa de mortalidade em todo o mundo e acomete aproximadamente 16% da população da cidade de São Paulo.^(^ 15 ^)^ Um estudo demonstrou que a DPOC é subdiagnosticada nessa cidade, pois 83% dos indivíduos com obstrução das vias aéreas não tinham diagnóstico clínico de DPOC.^(^ 16 ^)^ Esse cenário é mantido de forma praticamente inalterada, como foi constatado em um estudo de seguimento de 9 anos, o qual verificou que 70% de novos entrevistados apresentavam obstrução diagnosticada por espirometria.^(^ 16 ^)^ Além da destruição dos septos alveolares, a DPOC parece ter uma expressão sistêmica de caráter inflamatório.^(^ 17 ^)^ Possivelmente, essa inflamação atinge também o sistema endotelial,^(^ 7 ^)^ cuja implicação poderia, em parte, explicar a alta prevalência de doenças vasculares em pacientes com DPOC. Alguns estudos têm tentado relacionar o aumento dos níveis de alguns marcadores endoteliais, como proteína C reativa e fibrinogênio, com a DPOC.^(^ 7 ^,^ 8 ^)^ Há um relato de aumento do FvW em pacientes com DPOC durante o período de exacerbação.^(^ 10 ^)^ Entretanto, o papel desse marcador na DPOC durante a fase estável da doença ainda não foi estabelecido. O FvW pode ser avaliado de duas formas distintas: pelo seu valor sérico absoluto e pelo seu percentual de atividade sérica. A primeira é uma avaliação quantitativa, enquanto a segunda nos remete a uma análise qualitativa.

No atual estudo, os autores encontraram um valor sérico absoluto de FvW elevado nos tabagistas (com e sem obstrução ao fluxo aéreo) quando comparados ao grupo controle (p < 0,01). A relação entre o tabagismo e o aumento dos níveis do FvW vem sendo demonstrada ao longo dos últimos anos, sendo que parece haver um incremento significativo, de até 76%, após 120 minutos do uso de cigarro, assim como uma redução média de 144% para 123% nos níveis do FvW em pacientes que param de fumar.^(^ 8 ^)^ Um estudo demonstrou que a atividade do FvW encontra-se aumentada em fumantes.^(^ 18 ^)^ Com relação a pacientes com DPOC, um grupo de autores reportou que o FvW está elevado nesses pacientes quando comparados a indivíduos saudáveis; no entanto, pacientes tabagistas sem obstrução não foram incluídos naquela análise.^(^ 19 ^)^ Em outro estudo, foi demonstrado que os níveis séricos do FvW aumentam em pacientes com DPOC com exacerbação.^(^ 20 ^)^ No presente estudo, a presença de exacerbação foi considerada um critério de exclusão, pois objetivamos analisar os níveis de FvW durante a fase estável da doença obstrutiva, e portanto não foi possível verificar tal associação. Os autores verificaram que o percentual de atividade do FvW em pacientes com obstrução brônquica foi significativamente superior quando comparado com tabagistas sem obstrução (p < 0,05). O aumento em percentual da atividade do FvW em pacientes com DPOC, quando comparados ao grupo de tabagistas, sugere o papel do FvW na fisiopatologia inflamatória do DPOC e sua correlação com aterosclerose e doença cardiovascular.^(^ 7 ^)^

Até onde sabemos, o presente estudo é o primeiro a tentar correlacionar os níveis do FvW com a gravidade da DPOC conforme a classificação GOLD.^(^ 14 ^)^ Entretanto, não foi encontrada nenhuma diferença estatisticamente significativa entre os quatro grupos de gravidade com relação aos níveis séricos do FvW. Tampouco observamos diferenças entre os grupos quando foi considerada a classificação espirométrica da DPOC. Isso sugere que, apesar de os níveis do FvW estarem elevados em pacientes com DPOC estáveis, esses não se correlacionam com a gravidade da doença. Tal achado é compatível com alguns relatos na literatura que relacionam os níveis do FvW com outras doenças inflamatórias, como diabetes mellitus e artrite reumatoide.^(^ 10 ^,^ 21 ^)^ O FvW parece ser um marcador inespecífico de inflamação, não se prestando a graduar a gravidade de doenças inflamatórias crônicas.

Analisando a presença de sintomas através da escala de MRCm, foram observados valores do percentual de atividade do FvW significativamente maiores em pacientes mais sintomáticos, ou seja, aqueles com MRCm ≥ 2 (p < 0,05). Esse fato possivelmente indica que pacientes sintomáticos apresentem um grau de inflamação maior do que aqueles assintomáticos ou pouco sintomáticos. Nessa linha de raciocínio, era de se esperar que pacientes com exacerbações frequentes apresentassem níveis mais elevados de FvW, o que não foi observado na presente amostra. Dessa forma, são necessários mais estudos para elucidar esse achado.

Embora tenha havido uma correlação negativa significativa entre os valores de VEF_1_ em porcentagem do previsto e os níveis percentuais de atividade do FvW em todos os três grupos (controle, tabagista e DPOC), essa correlação foi pouco robusta (Figura 3), e um estudo não encontrou correlações entre os níveis do FvW e o declínio de VEF_1_.^(^ 22 ^)^ Portanto, estudos com um número maior de pacientes são necessários para corroborar esse achado.

O presente estudo apresenta algumas limitações. Entre elas, destaca-se o não pareamento dos grupos controle e tabagista com o grupo DPOC no quesito idade, fato esse sempre muito difícil de ser conseguido em estudos que comparam pacientes com e sem obstrução brônquica. No entanto, os voluntários saudáveis (controles) e os tabagistas tinham idades semelhantes. Mesmo assim, os valores de FvW foram significativamente mais elevados no grupo de fumantes. Outro dado importante é que não foi avaliado o grupo sanguíneo (sistema ABO) dos pacientes, que apresenta uma pequena influência nos níveis do FvW. Uma terceira limitação foi a não avaliação de outros parâmetros inflamatórios, como proteína C reativa e fibrinogênio, o que nos permitiria compará-los com dados da literatura relacionados a esses marcadores e com os níveis séricos do FvW. Por outro lado, houve uma tentativa de excluir um grande número de fatores que poderiam estar relacionados à inflamação sistêmica e à lesão endotelial. Assim, conforme relatado na metodologia, pacientes ou voluntários com histórico de doença cardiovascular ou de outras doenças crônicas ou infecciosas, assim como aqueles que estavam em uso de medicamentos, foram excluídos do estudo, fato esse que limitou bastante a entrada de participantes.

Pacientes com DPOC apresentam um maior risco de lesão endotelial e consequente doença cardiovascular. Em nosso estudo, os níveis séricos absolutos do FvW estavam aumentados em indivíduos tabagistas com e sem obstrução brônquica quando comparados aos controles, e o percentual de atividade do FvW estava aumentado nos pacientes com DPOC quando comparados aos tabagistas. É possível que o FvW participe do processo inflamatório sistêmico em pacientes com DPOC e, dessa forma, contribua com o aumento do risco cardiovascular.
